# Ultrastructure of GABA- and Tachykinin-Immunoreactive Neurons in the Lower Division of the Central Body of the Desert Locust

**DOI:** 10.3389/fnbeh.2016.00230

**Published:** 2016-12-06

**Authors:** Uwe Homberg, Monika Müller

**Affiliations:** ^1^Faculty of Biology, Animal Physiology, Philipps-UniversitätMarburg, Germany; ^2^Institute for Zoology, University of RegensburgRegensburg, Germany

**Keywords:** insect brain, central complex, γ-aminobutyric acid, locustatachykinin, synaptic organization, desert locust

## Abstract

The central complex, a group of neuropils spanning the midline of the insect brain, plays a key role in spatial orientation and navigation. In the desert locust and other species, many neurons of the central complex are sensitive to the oscillation plane of polarized light above the animal and are likely involved in the coding of compass directions derived from the polarization pattern of the sky. Polarized light signals enter the locust central complex primarily through two types of γ-aminobutyric acid (GABA)-immunoreactive tangential neurons, termed TL2 and TL3 that innervate specific layers of the lower division of the central body (CBL). Candidate postsynaptic partners are columnar neurons (CL1) connecting the CBL to the protocerebral bridge (PB). Subsets of CL1 neurons are immunoreactive to antisera against locustatachykinin (LomTK). To better understand the synaptic connectivities of tangential and columnar neurons in the CBL, we studied its ultrastructural organization in the desert locust, both with conventional electron microscopy and in preparations immunolabeled for GABA or LomTK. Neuronal profiles in the CBL were rich in mitochondria and vesicles. Three types of vesicles were distinguished: small clear vesicles with diameters of 20–40 nm, dark dense-core vesicles (diameter 70–120 nm), and granular dense-core vesicles (diameter 70–80 nm). Neurons were connected via divergent dyads and, less frequently, through convergent dyads. GABA-immunoreactive neurons contained small clear vesicles and small numbers of dark dense core vesicles. They had both pre- and postsynaptic contacts but output synapses were observed more frequently than input synapses. LomTK immunostaining was concentrated on large granular vesicles; neurons had pre- and postsynaptic connections often with neurons assumed to be GABAergic. The data suggest that GABA-immunoreactive tangential neurons provide signals to postsynaptic neurons in the CBL, including LomTK-immunolabeled CL1 neurons, but in addition also receive input from LomTK-labeled neurons. Both types of neuron are additionally involved in local circuits with other constituents of the CBL.

## Introduction

**Figure 1:**
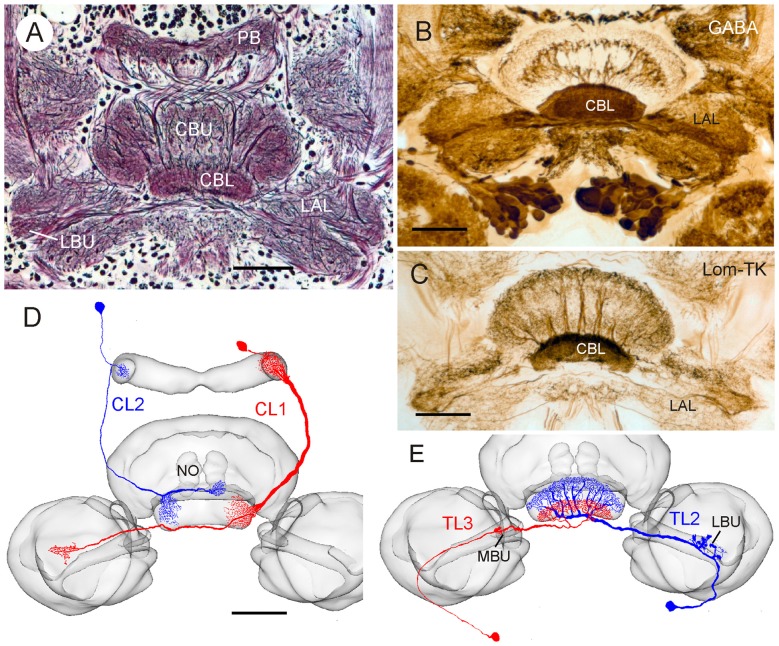
**Anatomical and neurochemical organization of the lower division of the locust central body (CBL). (A)** Frontal Bodian-stained paraffin section through the central complex and lateral complexes. CBL, lower division of the central body; CBU, upper division of the central body; LAL, lateral accessory lobe, LBU, lateral bulb; PB, protocerebral bridge. **(B)** Frontal Vibratome section illustrating dense γ-aminobutyric acid (GABA) immunolabeling in the CBL, revealed by the peroxidase-antiperoxidase (PAP) technique as described by Homberg et al. (1999). **(C)** Immunostaining of the CBL on frontal Vibratome section using an antiserum against locustatachykinin II (LomTK II); PAP technique as described by Vitzthum and Homberg (1998). **(D,E)** Two types of columnar **(D)** and tangential **(E)** neuron innervating the CBL. Frontal camera lucida reconstructions of Neurobiotin- or Lucifer Yellow-labeled neurons were projected onto the standard locust central complex (el Jundi et al., 2010). **(D)** Columnar neuron 1 and 2 (CL1, CL2). NO, nodulus. **(E)** Tangential neuron 2 and 3 (TL2, TL3). MBU, medial bulb. Scale bars: 100 μm.

The central complex comprises a group of neuropils in the insect brain that extend across the brain midline. Prominent subdivisions are the protocerebral bridge (PB), the upper (CBU) and lower (CBL) divisions of the central body, also termed fan-shaped body and ellipsoid body, respectively, and a pair of globular noduli (Figure 1A; Ito et al., 2014; Pfeiffer and Homberg, 2014). The PB, the CBL and the CBU are subdivided into linear arrangements of 16 slices (in *Drosophila* 18), and numerous sets of columnar neurons provide intricate chiasmal connections between the slices of the different subcompartments (Figure 1A; Heinze and Homberg, 2008; Wolff et al., 2015). Convergent evidence from studies in flies, beetles, the monarch butterfly, the desert locust, the honeybee, and the field cricket point to a role for the central complex in spatial orientation. In fruit flies, the central complex is involved in spatial working memory and place learning (Neuser et al., 2008; Ofstad et al., 2011). Calcium imaging in tethered walking *Drosophila* revealed a 360° representation of headings in columnar neurons of the ellipsoid body (Seelig and Jayaraman, 2015). Likewise, extracellular recordings from central-complex neurons of the discoid cockroach demonstrated head-direction coding (Varga and Ritzmann, 2016). In dung beetles, the field cricket, the desert locust and the monarch butterfly, neurons of the central complex are sensitive to the plane of dorsally presented polarized light and likely signal compass directions provided by the polarization pattern of the blue sky (Homberg et al., 2011; Heinze, 2014; el Jundi et al., 2015). In the desert locust zenithal *E*-vectors are topographically represented in the slices of the PB, indicating a compass-like representation of celestial directions (Heinze and Homberg, 2007).

Photoreceptors in a specialized dorsal rim area of the compound eye are sensitive to the oscillation plane of celestial polarized light (Labhart and Meyer, 1999; Schmeling et al., 2014, 2015). Signals are transferred via a specific pathway to the CBL (Homberg et al., 2003; Pfeiffer and Kinoshita, 2012; Held et al., 2016; Schmitt et al., 2016). In the desert locust, three types of tangential neuron to the CBL, termed TL1, TL2, and TL3 neurons provide polarization signals to the central complex (Vitzthum et al., 2002; Heinze et al., 2009). Two of these cell types, TL2 and TL3, comprising as many as 100 bilateral pairs of neurons, are immunoreactive to antisera against γ-aminobutyric acid (GABA; Figures 1B,E; Homberg et al., 1999). The neurons receive massive dendritic input in microglomerular synaptic complexes from presynaptic projection neurons of the anterior optic tubercles (Träger et al., 2007). Heinze and Homberg (2009) suggested that TL neurons synapse upon certain types of columnar neurons (CL neurons, Figure 1D) in the CBL, which would carry the polarization signal to the PB, but synaptic connectivities in the CBL have so far only been inferred from the light microscopic appearances of terminal arborizations of neurons in the CBL. A subpopulation of CL neurons of the locust is immunoreactive to antisera against the neuropeptide locustatachykinin (LomTK; Figure 1C; Vitzthum and Homberg, 1998). To further elucidate the synaptic organization at the input stage to the polarization vision network in the central complex, we investigated the ultrastructural organization of the CBL in the desert locust and analyzed the connectivities of GABA- and LomTK-immunoreactive neurons.

## Materials and Methods

### Animals

Experiments were performed on adult locusts, *Schistocerca gregaria*, obtained from crowded colonies at the University of Regensburg. Animals were reared under 12L:12D photoperiod, and a temperature of 34°C during the light phase and 27°C during the dark phase. Experiments were performed on sexually mature adult males and females. Prior to dissection animals were immobilized by cooling to 4°C.

### Conventional Electron Microscopy

For routine electron microscopy, dissected brains were fixed for 4 h or overnight in freshly prepared fixative containing 2% paraformaldehyde/2.5% glutaraldehyde in sodium cacodylate buffer (0.1 M, pH 7.2), and 2% sucrose. After rinsing in sodium cacodylate buffer, brains were postfixed in osmium tetroxide (1% in sodium cacodylate buffer) for 1 h, dehydrated in an ethanol series and embedded in Epon 812 (Serva, Heidelberg, Germany) or Durcupan (Fluka, Buchs, Switzerland). Ultrathin sections (~70 nm in thickness, gray to silver interference colors) were cut in a frontal plane on an ultramicrotome (Ultracut E, Reichert, Vienna, Austria) and collected on copper slot grids coated with pioloform (Plano, Marburg, Germany). Sections were contrasted with 2% uranyl acetate (15 min) followed by lead citrate (5 min; Venable and Coggeshall, 1965).

### GABA Immunolabeling, Preembedding Technique

GABA immunolabeling on ultrathin sections was performed following the preembedding technique and the postembedding technique (immunogold technique). Two antisera against GABA were employed. Anti-GABA antiserum I was raised in rabbit against conjugates of GABA and keyhole limpet hemocyanin (#9/24, provided by Dr. T.G. Kingan). The antiserum has been widely used to study GABA immunostaining at the light microscopic level in insect nervous systems (Hoskins et al., 1986; Homberg et al., 1987, 1999). The anti-GABA antiserum II (#4 TB) was provided by Dr. H. Dircksen. The antiserum was raised in rabbits against GABA-glutaraldehyde conjugates as described by Seguela et al. (1984). In the locust brain and central complex both antisera revealed virtually identical immunolabeling patterns (Homberg et al., 1999). They provided dense immunostaining throughout the CBL (Figure 1B) resulting from immunolabeled TL2 and TL3 neurons, whereas TL1 neurons were immunonegative (Homberg et al., 1999). Preadsorption of the diluted antiserum I with 15 nM GABA-BSA (bovine serum albumin), and the diluted antiserum II with 20 μM GABA-glutaraldehyde conjugate abolished all immunolabeling on 30-μm microtome sections (Homberg et al., 1999). The GABA antiserum II has, in addition, been used in immunogold labeling of ultrathin sections of the locust brain (Träger et al., 2007). Here, omission of the primary antiserum abolished all staining, and preadsorption of the diluted antiserum with 20 μM GABA-glutaraldehyde conjugate again resulted in complete loss of immunostaining (Träger et al., 2007).

For preembedding immunolabeling, brains were dissected and immersed for 45 min in fixative containing 2.5% glutaraldehyde in 0.1 M sodium phosphate buffer (pH 7.4). Brains were then embedded in gelatin/albumin. Frontal sections at 20 μm were cut with a Vibratome (Technical Products, St. Louis, MO, USA) and collected in cell culture plates. GABA immunostaining was performed on the free floating sections following the peroxidase-antiperoxidase (PAP) technique of Sternberger (1979). To reduce background staining, the sections were preincubated for 1 h in 10 mM phosphate buffered saline (PBS) containing 0.01% Triton X-100 (TrX) and 10% normal goat serum (NGS). Incubation in anti-GABA antiserum I, diluted 1:4000 or antiserum II, diluted 1:20,000 in 10 mM PBS, 0.01% Triton X-100 and 1% NGS was performed overnight at 4°C. Following rinses in 10 mM PBS, 0.01% TrX and 1% NGS, the sections were incubated for 2 h in secondary antiserum, goat anti-rabbit (1:80 in 10 mM PBS, 0.01% TrX and 1% NGS; Sigma, Deisenhofen, Germany). Afterwards, the sections were rinsed again and incubated for 2 h in rabbit PAP complex (1:100 in 10 mM PBS, 0.01% TrX and 1% NGS; Dakopatts, Hamburg, Germany). Subsequently the sections were rinsed 3 × 10 min in 0.05 M Tris/HCl (pH 7.4) and stained in 0.02% 3,3-diaminobenzidine tetrahydrochloride (Sigma) in 0.05 M Tris/HCl. The redox reaction was started by adding 10 μl H_2_O_2_ (30% solution) per 50 ml solution. The development of the brown reaction product was controlled visually and stopped by rinses in 0.05 M Tris/HCl.

For ultrastructural analysis, the sections were transferred to 10 mM phosphate buffer (pH 7.4) and postfixed for 20–30 min in 1% OsO_4_ in phosphate buffer. Following rinses in phosphate buffer, the sections were dehydrated in an ascending ethanol series and embedded in Epon 812 as described above. The sections were attached to an Epon block, sectioned with an Ultracut microtome and contrasted as described above.

### GABA Immunolabeling, Postembedding Technique

For immunogold labeling (postembedding staining), brains were dissected and fixed as described for conventional electron microscopy. Brains were embedded in Araldite epoxy resin (EMS, Washington, PA, USA). Ultrathin sections were collected on coated nickel slot grids. Prior to immunolabeling, ultrathin sections were incubated for 1 h on drops of a 25% aqueous solution of sodium metaperiodate. After thorough washing in distilled water and 0.5 M HCl, grids were incubated in PBS containing 0.01% TrX for 15 min and subsequently for 30 min in incubation buffer (0.01 M sodium phosphate buffer, pH 7.4, containing 0.5 M NaCl, 0.75% fish gelatin, 0.1% ovalbumin, and 0.01% Tween 20). The sections were exposed to the anti-GABA antiserum I (1:6000), or anti-GABA antiserum II (1:2000–1:6000) in incubation buffer for at least 15 h at 4°C and were afterwards washed thoroughly in PBS. Finally, the grids were transferred for 4 h at room temperature to a solution of 10-nm gold-labeled goat-anti-rabbit antibody (Auro Probe EM GAR G10, Amersham, Braunschweig, Germany) diluted 1:40 in incubation buffer. Preparations were then washed in PBS and distilled water and were contrasted with uranyl acetate (8 min) and lead citrate (2 min). In control experiments, substitution of the primary antibody solution with incubation buffer resulted in complete lack of gold labeling. Preadsorption of the GABA antiserum with 20 μM GABA-glutaraldehyde conjugate, prepared as described by Ottersen et al. (1986), abolished all immunostaining.

### LomTK Immunolabeling, Postembedding Technique

Brains were dissected and fixed as described for conventional electron microscopy. After postfixation in OsO_4_ and dehydration as described above they were embedded in Epon 812 or Durcupan. Immunolabeling was performed as described for GABA-immunogold staining. The anti-LomTK II antiserum (kindly provided by Dr. Hans Agricola, Jena) was used at a concentration of 1:1000. The antiserum has been used to analyze LomTK immunostaining on Vibratome sections of the locust central complex at the light microscopic level (Vitzthum and Homberg, 1998). In that study, preadsorption of the primary antiserum with 10 μM LomTK II abolished all staining.

### Data Evaluation and Statistics

Ultrathin sections were examined and photographed with a transmission electron microscope (EM 109 and EM10C, Zeiss, Oberkochen, Germany). Photomicrographs (Agfa Scientia EM film) from selected sections were scanned at 2400 dpi (CanoScan 9000F MarkII, Canon, Tokyo, Japan) and processed further with Adobe Photoshop CS3 and CorelDRAW X3.

GABA-labeled immunogold sections were evaluated statistically. The mean labeling density of gold particles (GPs) over selected profiles was determined in 4–8 consecutive ultrathin sections and compared against GP levels in adjacent, presumably unlabeled profiles of similar size and organelle composition (Watson, 1988; Watson et al., 2000; Träger et al., 2007). Profiles were accepted as GABA-immunoreactive, if they had a significantly higher GP concentration than an adjacent reference profile (*t*-test, *p* < 0.05); when tested against two adjacent profiles, one-way ANOVA with Tukey-HSD multiple range test was performed (*p* < 0.05).

## Results

The anatomical organization and patterns of GABA- and LomTK immunostaining in the lower division of the locust central body (CBL) are shown in Figure 1. All layers of the CBL exhibit dense GABA immunolabeling (Figure 1B). Homberg et al. (1999) showed that two types of tangential neuron, termed TL2 and TL3 (Figure 1E) are GABA-immunoreactive. TL2 and TL3 neurons have ramifications in microglomeruli of the lateral (TL2), resp. medial bulb (MBU; TL3). They connect the bulbs to all layers of the CBL, but individual neurons of both types only invade specific layers of the CBL. In addition, two major types of columnar neuron with ramifications in the CBL, termed CL1 and CL2 have been identified (Figure 1D; Müller et al., 1997). Both types of CL neuron provide connections between slices of the CBL and the PB (Figure 1D). Whereas most types of CL1 neurons have additional ramifications in small areas of the lateral accessory lobe (LAL), likely homologous to the gall in *Drosophila*, TL2 neurons have fine projections in the lower unit of a nodulus (Figure 1D). Vitzthum and Homberg (1998) showed that two subtypes of CL1 neurons exhibit immunostaining with antisera against LomTK resulting in dense immunolabeling of the CBL (Figure 1C).

### Ultrastructural Organization of the CBL

**Figure 2:**
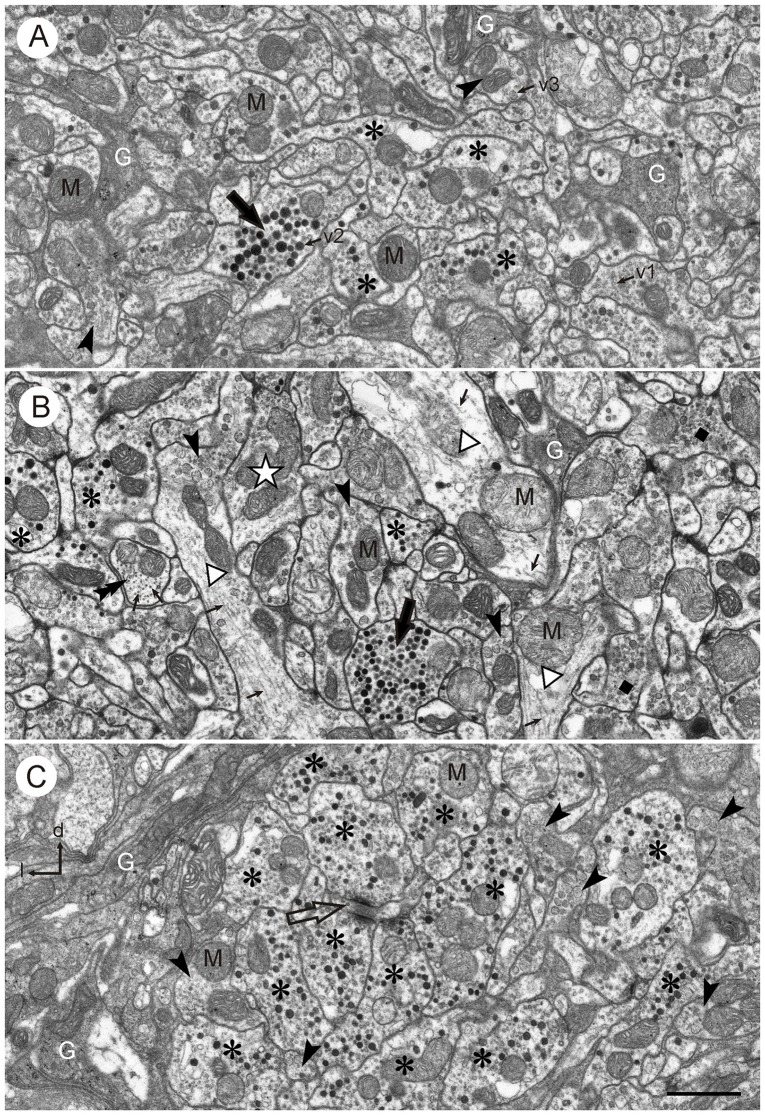
**Ultrastructure of the CBL. (A,B)** Central neuropil area from two different brains. The CBL consists of densely packed neuronal processes containing mitochondria (M) and numerous vesicles (small arrows; v1: small clear vesicles; v2: large dense core vesicles, v3: large granular vesicles). Glial processes (G) extend between the neuronal profiles. Neuronal profiles differ with respect to vesicle composition. Profiles with small clear vesicles and dark dense core vesicles (asterisks) and profiles with large granular vesicles (arrowheads) occur frequently. Profiles with numerous dense core vesicles (large black arrows) and profiles with small clear and granular vesicles (dark squares in **B**) occur more rarely. The white star in **(B)** marks a profile which contains only clear vesicles. Three large neuronal processes in (**B**; open triangles) contain only few vesicles but numerous microtubules (small arrows). The double arrow in **(B)** points to a cross-sectioned fiber which contains no vesicles but numerous cross-sectioned microtubules. **(C)** Section from the left dorsal margin of the CBL, same brain as **(B)**. It shows an accumulation of profiles with small clear and large dense core vesicles (asterisks). Two of these profiles make output synapses onto a smaller third profile (open arrow). Near this cluster of profiles are some processes with large granular vesicles (arrowheads). The dorso-lateral margin of the CBL is covered with layers of glial processes (G). l, left; d, dorsal. Scale bar in **(C)**: 1 μm (applies to **A–C**).

At the ultrastructural level, neuronal profiles of different size, shape and organelle composition could be distinguished in the CBL. Glial processes were interspersed between neuronal profiles, and especially large neuronal fibers were surrounded by glia (Figure 2). Although no precise measurements were done, in all sections studied the proportion of glia in the neuropil was considerably lower than that of neuronal profiles. The CBL as a whole was largely surrounded by a glial sheath against adjacent neuropils (Figure 2C) but along its dorsal face, a glial boundary against the adjacent CBU was only partially present.

**Figure 3:**
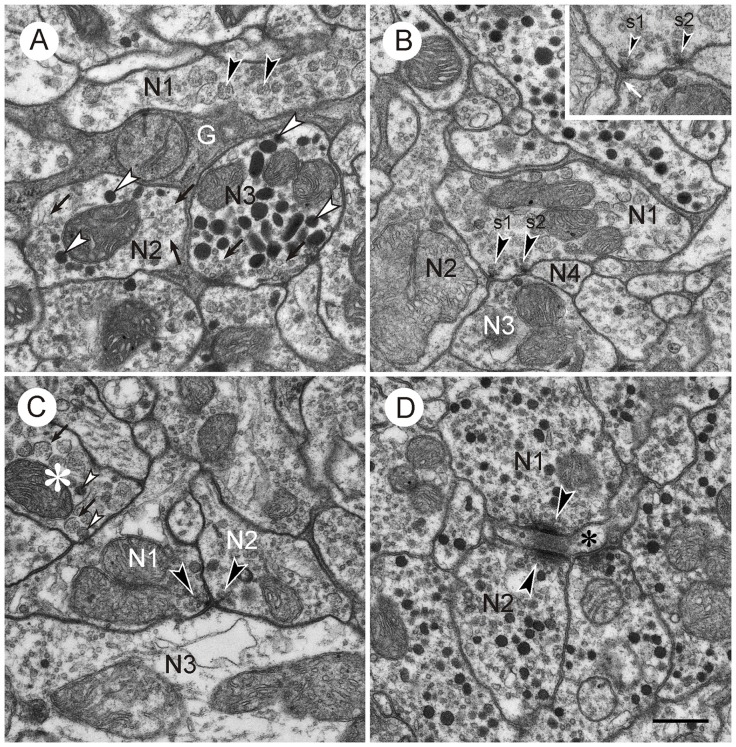
**Vesicles and synapses of neuronal profiles in the CBL.** Details from frontal ultrathin sections through the CBL. **(A)** Central neuropil area showing neuronal profiles with different vesicle content (N1–N3) and processes from a glial cell (G). N1, neuronal profile with large granular vesicles (arrowheads); N2, neuronal profile with small clear pleomorphic vesicles and large dense core vesicles; N3, profile with circular small clear vesicles (arrows) and large ovoid dense core vesicles (white arrowheads). Note the different appearance of the small vesicles in N2 and N3. **(B)** Central neuropil area. Neuronal profile with large granular vesicles (N1) and two dyadic output synapses (s1, s2, arrowheads). Both synapses are of type I, i.e., one presynaptic profile (N1) faces two postsynaptic profiles (N2 and N3, resp. N3 and N4). Inset shows synaptic profiles at higher magnification (45,000×). Both synapses show a presynaptic membrane density (arrowheads) surrounded by synaptic vesicles. The intercellular space in enlarged at the active zone. The postsynaptic membrane only shows minor accumulation of electron dense material (white arrow). **(C)** Type II synapse. Two presynaptic profiles (N1,N2) with small clear synaptic vesicles and large granular vesicles face a postsynaptic profile with small clear and large dense core vesicles. Arrowheads point to presynaptic densities with accumulations of clear synaptic vesicles. The asterisk marks a profile with large granular vesicles. The membrane of most vesicles is broken (arrows). These have a lighter granular content than intact vesicles (white arrowheads). **(D)** Magnified detail from Figure 2C. Profiles N1 and N2 with small clear and dark dense core vesicles are presynaptic to a third small profile (asterisk). In adjacent sections, this profile contained large granular vesicles. Presynaptic structures consist of a bar-shaped density surrounded by small clear vesicles. The synapses may be monadic or dyadic. Scale bar in **(D)**: 0.5 μm (applies to **A–D**).

Neuronal profiles in the CBL were rich in mitochondria and contained numerous vesicles. Three main types of vesicles could be distinguished: (1) small, clear vesicles (Figures 2A–C, 3); they had a circular or ovoid form and a size of 20–40 nm; (2) dense-core vesicles filled with homogeneous electron dense material; they usually had a spheroidal form and a size of 70–120 nm, but some profiles also contained elongated ovoid dense core vesicles (Figure 3A); and (3) dense-core vesicles filled with granular material (Figures 2, 3C). In most cases their membranes were ruptured, but when intact they had diameters of 70–80 nm (Figures 2, 3C). Their content was granular and more lightly stained than that of type 2 dense core vesicles. All numbers for vesicle sizes are based on uncorrected measurements of vesicle profiles taken from the sections and may, thus, slightly underestimate the true range of diameters, because profiles sectioned peripherally are likely included in these numbers. The distinction of three vesicle types is based on obvious differences in size and electron density, but it is likely that further subtypes may be distinguished based on statistical evaluation of size and shape. Especially the small clear vesicles appeared to be rather pleomorphic and indistinct in some profiles, whereas in others they were more distinct and uniform in size and shape. Likewise, the granular content of type 3 vesicles may be further differentiated based on electron density and granular appearance.

Many neuronal profiles of the CBL contained two different types of vesicles. Based on vesicle composition, the following types of profiles, resp. neurons could be distinguished:

1. Profiles with many small clear vesicles of pleomorphic appearance and usually smaller numbers of dense core vesicles (Figures 2, 3A,D). These profiles occurred most frequently in the CBL. Figure 2C shows a section through the left dorsal margin of the CBL with numerous profiles of this type.
2. Profiles with granular dense-core vesicles. These profiles were again abundant in the CBL (Figures 2A–C, 3A–C).
3. Profiles densely filled with dark dense core vesicles of spheroidal or ovoid shape (Figures 2A,B, 3A). These profiles usually also contained small clear vesicles (Figure 3A). These profiles occurred regularly but more rarely than the previous two types.
4. Profiles with small clear vesicles and granular vesicles (Figure 2B). These profiles were found rarely.

In some cases, sectioned profiles only showed small clear vesicles (e.g., Figure 1B). In adjacent sections, however, other types of vesicles were usually seen in addition in these profiles. Therefore, it remains unclear whether profiles exist that contain exclusively small clear vesicles. Some profiles did not contain vesicles. Those profiles were either very small (0.03–0.05 μm^2^) or quite large. The large profiles contained microtubules, suggesting that they were larger neurites or axonal profiles. Figure 2B shows several large profiles which in large parts do not contain vesicles but microtubules. Those profiles were probably neural fibers extending to terminal, vesicle containing ramifications.

### Synaptic Contacts in the CBL

**Figure 4:**
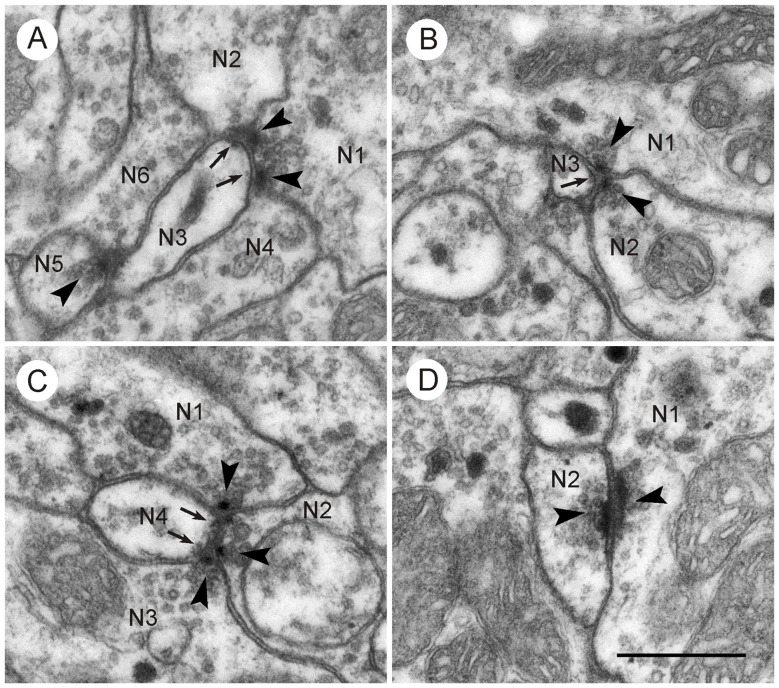
**Synaptic contacts in the CBL.** Details from frontal sections through the CBL from a brain embedded in Durcupan. **(A)** Type I synapses. N1 is presynaptic to N2/N3 and to N3/N4. N5 is also presynaptic to N3 and N6. Arrowheads point to synaptic densities and vesicles. Arrows point to indistinct postsynaptic densities in N3. **(B)** Type II synapse. The profiles N1 and N2 show typical presynaptic characters (arrowheads). They face a third postsynaptic profile, N3. Arrows point to postsynaptic densities in N3. **(C)** Type I and type II synapse. Profile N4 receives triple synaptic input via a type I synapse (N1 presynaptic to N2 and N4) and a type II synapse (N2 and N2 presynaptic to N4). Arrowheads point to presynaptic specializations, arrow to minor postsynaptic density. **(D)** Two profiles (N1,N2) with bar-shaped presynaptic densities (arrowheads) facing each other. It may be a type II synapse which has been sectioned such that only the two presynaptic profiles are visible. Scale bar in **(D)**: 0.5 μm (applies to **A–D**).

Synaptic contacts in the CBL were identified according to established criteria for insect nervous tissue, such as a presynaptic zone with accumulation of synaptic vesicles and electron dense material at the presynaptic membrane; a synaptic cleft, characterized by electron dense material in an enlarged intercellular space between pre- and postsynaptic membrane; and more or less prominent accumulation of electron dense material along the postsynaptic membrane (Steiger, 1967; Boeckh et al., 1970; Schürmann and Wechsler, 1970; Dowling and Chappell, 1972; Goodman et al., 1979; Tolbert and Hildebrand, 1981; Watson and Burrows, 1982). In the CBL, synaptic contacts between profiles were abundant. With few exceptions (see below) three profiles contributed to a synaptic figure. In most cases (type I synapse), one presynaptic profile was opposite two postsynaptic profiles. This configuration, a synaptic dyad, has been described in many studies before Westfall (1987). At the contact area of the three profiles, accumulation of electron dense material occurred along the presynaptic profile, opposite from the two postsynaptic profiles (Figures 2B, 3A–C). This aggregation had a round to triangular shape and was usually surrounded by small clear vesicles. Dense core vesicles were never observed near this area. The intercellular cleft between the three profiles was enlarged and filled with moderately electron dense material. Accumulation of electron dense material along the postsynaptic membranes was sparser or completely absent (Figures 3B, 4A–C).

In addition to type I synaptic profiles, numerous synapses were found in which two profiles with presynaptic structures were opposed to one postsynaptic profile (type II synapse; Figures 3C, 4B,C). In both of these synaptic configurations, the presynaptic characters were observed across two or more ultrathin sections. Therefore, the round or triangularly shaped presynaptic density rather has the three-dimensional form of an elongated bar which is typical for locust central synapses (Watson and Schürmann, 2002). In some cases, these densities were sectioned longitudinally, revealing their bar-shaped form (Figures 3D, 4D). The elongated presynaptic densities were associated with clear, translucent vesicles along their entire length. It is possible that the synapses in Figure 4D were monadic, consisting of one presynaptic and one postsynaptic profile, but in this case, the adjacent critical section, which would have revealed whether a third profile is present, was not available. Therefore, the existence of monadic synapses cannot be excluded in the CBL, but the large majority of synaptic profiles had a dyadic symmetry when cut in cross section. Besides the described types of synapses, no evidence for other mechanisms of neuronal communication was found, such as exocytosis of dense core vesicles or structures suggesting electrical synapses.

### Ultrastructure of GABA-Immunoreactive Profiles

**Figure 5:**
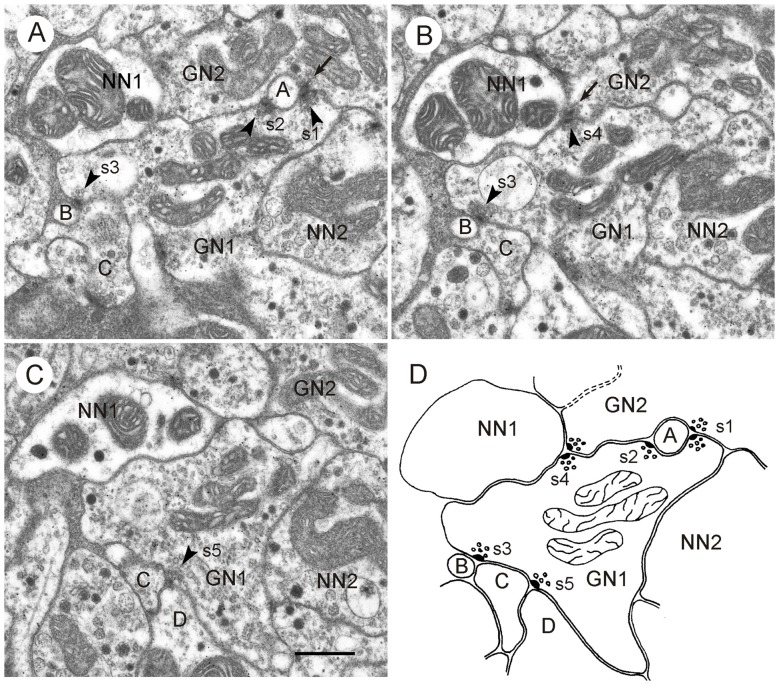
**GABA-immunoreactive neuron with output synapses. (A–C)** Details from section three **(A)**, five **(B)** and eight **(C)** out of eight consecutive immunogold-labeled sections through the CBL. The GABA-immunoreactive profile (GN1) makes five output synapses (s1-s5) to neighboring profiles (**A–D**, NN1). Arrowheads point to presynaptic densities. Two of these synapses (s1 in **A** and s4 in **B**) are of type II, i.e., the presynaptic profile (GN1) and a second presynaptic profile (GN2) face one postsynaptic profile (A and NN1). GN2 is like GN1 GABA-immunolabeled. The postsynaptic profile NN1 of s4 was not immunolabeled; it contains a few dense core vesicles. Whether profile A is GABA-immunolabeled or not, could not be determined. Output synapses s2, s3 and s5 are of type I. In synaptic profile s2, **(A)** GN1 is presynaptic to profile A and to the second GABA-immunolabeled profile GN2. In synaptic profiles s3 **(A,B)** and s5 **(C)** GN1 is presynaptic to profiles B and C (s3), resp. profiles C and D (s5). To the right of GN1, an unlabeled profile (NN2) contains granular vesicles. The mean gold particle (GP) densities (*n* = 8) were 13.0 ± 4.1 GP/μm^2^ (GN1), 8.0 ± 2.5 GP/μm^2^ (GN2) 1.8 ± 1.4 GP/μm^2^ (NN1), and 1.2 ± 0.9 GP/μm^2^ (NN2). The GP densities in GN1 and GN2 were significantly different from those in NN1 and NN2 (one-way ANOVA, Tukey-HSD, *p* < 0.05). **(D)** Schematic diagram illustrating synaptic contacts of GN1 in the eight sections. Scale bar in **(C)**: 0.5 μm (applies to **A–D**).

Both pre- and postembedding techniques yielded specific immunolabeling of neuronal profiles in the CBL and were used for analyzing synaptic connections. Control sections without primary antiserum were free of immunostaining. Owing to slight differences in GP densities, the GABA antiserum II resulted in superior immunogold labeling. Here, an antiserum dilution of 1:2000 yielded best staining quality and was used in most preparations evaluated in detail (Figures 5, 7). In areas outside the CBL, immunolabeled processes could be easily identified because they were surrounded by numerous unlabeled profiles. In the CBL, however, profiles with high GP densities were abundant, which made it more difficult to differentiate them from unlabeled neurons. We, therefore, evaluated GP densities in presumably immunolabeled profiles over 4–8 consecutive sections against neighboring, presumably unlabeled profiles of similar size. Profiles were accepted as GABA-immunoreactive, if GP densities were significantly different (*p* < 0.05) from those of neighboring presumably unlabeled profiles. In total, five analyzed profiles were evaluated. They had GP densities ranging from 8.0 ± 2.5 GP/μm^2^ to 18.4 ± 12.7 GP/μm^2^. Adjacent immunonegative profiles had GP densities ranging from 1.2 ± 0.9 GP/μm^2^ to 1.8 ± 1.4 GP/μm^2^. The ratio of GP densities between unlabeled and labeled profiles ranged from 1:6 to 1:11. Profiles that could neither be assigned as GABA-labeled nor unlabeled, e.g., because of small profile area, were termed “non-classified”.

**Figure 6:**
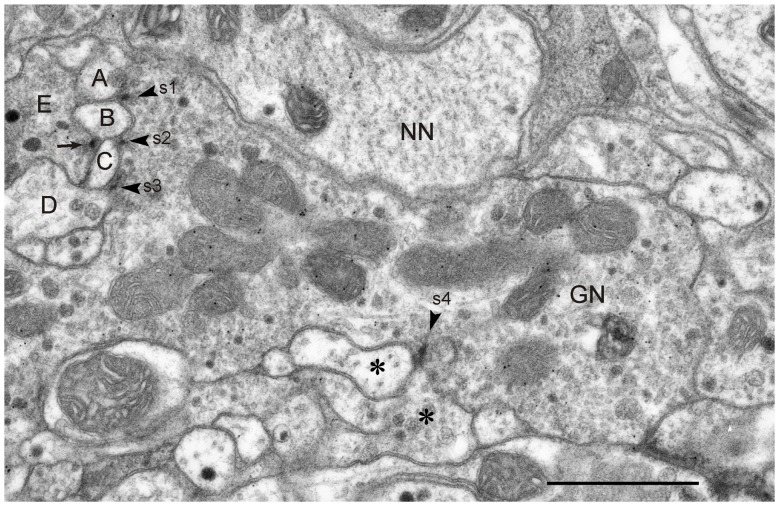
**GABA-immunoreactive neuron with output synapses.** Details from one out of four consecutive immunogold-labeled sections through the CBL near its dorsal boundary with the CBU. Dilution of the GABA antiserum 1:4000. The GABA-immunoreactive neuron (GN) makes four output synapses (s1-s4) with non-classified small profiles (A–D; asterisks) surrounding GN. Arrowheads point to presynaptic densities. All synapses are of type I. The small profiles B and C receive dual synaptic input. Both profiles are, in addition, postsynaptic to another probably also GABA-immunolabeled profile E. Arrow points to presynaptic density. One of the postsynaptic profiles at s3 and s4, respectively, contains granular dense core vesicles (D, right asterisk). The mean GP densities (*n* = 4) were 10.1 ± 4.0 GP/μm^2^ (GN), 1.3 ± 0.7 GP/μm^2^ for the profile NN, and 0.8 ± 0.7 GP/μm^2^ for a second unlabeled profile (not shown). The GP density in GN was significantly different from those of the two other profiles (one-way ANOVA, Tukey-HSD, *p* < 0.05). Scale bar: 1 μm.

**Figure 7:**
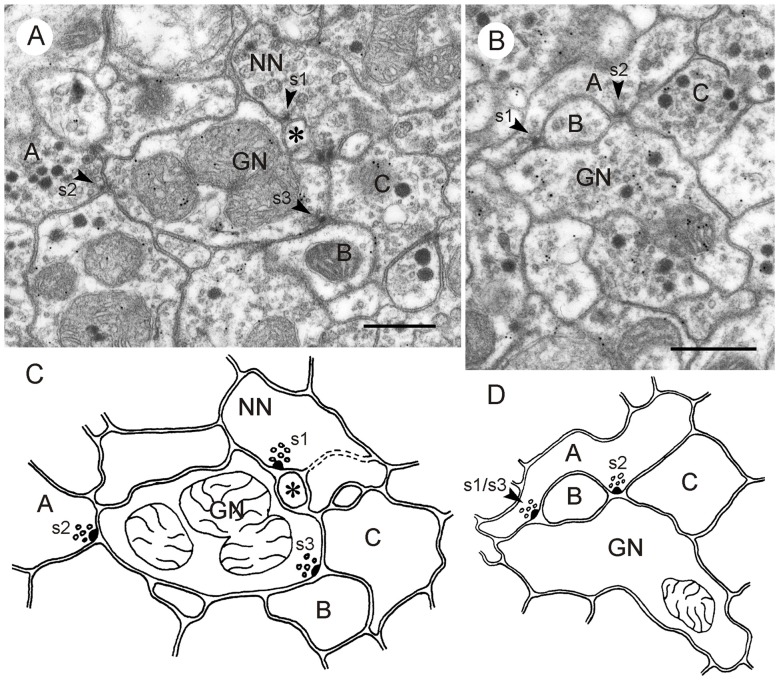
**GABA-immunoreactive neurons with input and output synapses. (A,C)** Section five out of five consecutive immunogold-labeled sections through the CBL **(A)** and corresponding schematic diagram of synaptic contacts **(C)**. A neuron with granular vesicles (NN) makes an input synapse of type I (s1, arrowhead) with the GABA-immunoreactive neuron and a second small profile (asterisk). Although NN had a relatively small diameter, it is most likely not immunolabeled because it contained no GP in any of the five sections. NN receives a second synaptic input from a profile with small clear and dark dense core vesicles (s2, arrowhead); its immunostaining could not be determined. Finally, GN is presynaptic to two other profiles (s3, B, C), illustrating serially synaptic connections of GN. The mean GP densities (*n* = 5) were 13.3 ± 6.0 GP/μm^2^ (GN), and for a second profile not shown 1.6 ± 1.4 GP/μm^2^. The GP densities in GN were significantly different from those of the second profile (*t*-test, *p* = 0.0031). **(B)** Section two out of five consecutive immunogold-labeled sections through the CBL. The GABA-immunoreactive profile receives dual synaptic input (s1, s2 arrowheads). In synapse s1, profile A is presynaptic to GN and a non-classified profile B (type I synapse). Synapse s2 might be a triadic synapse with one non-classified presynaptic profile (A) facing three postsynaptic profiles (GN, B, C). In the first section of this series, however (not shown), only profiles B and GN seemed to be postsynaptic to A. The mean GP densities (*n* = 5) were 18.4 ± 12.7 GP/μm^2^ (GN), and for a second profile with granular vesicles and small clear vesicles not shown 1.6 ± 1.5 GP/μm^2^. The large standard deviation of GP density of GN resulted from very low GP densities on one out of the five sections. Perhaps this section was not sufficiently exposed to the primary or secondary antibody (e.g., by an air bubble between section and incubation medium). The GP densities in GN were significantly different from those of the second profile (*t*-test, *p* = 0.02). **(D)** Schematic diagram of synaptic contacts of the series in **(B)**. Synapse s3 occurred on section 5 of the series at the same site as synapse s1, but with two sections (3 and 4) in between without synaptic contact. Scale bars: 0.5 μm.

**Figure 8:**
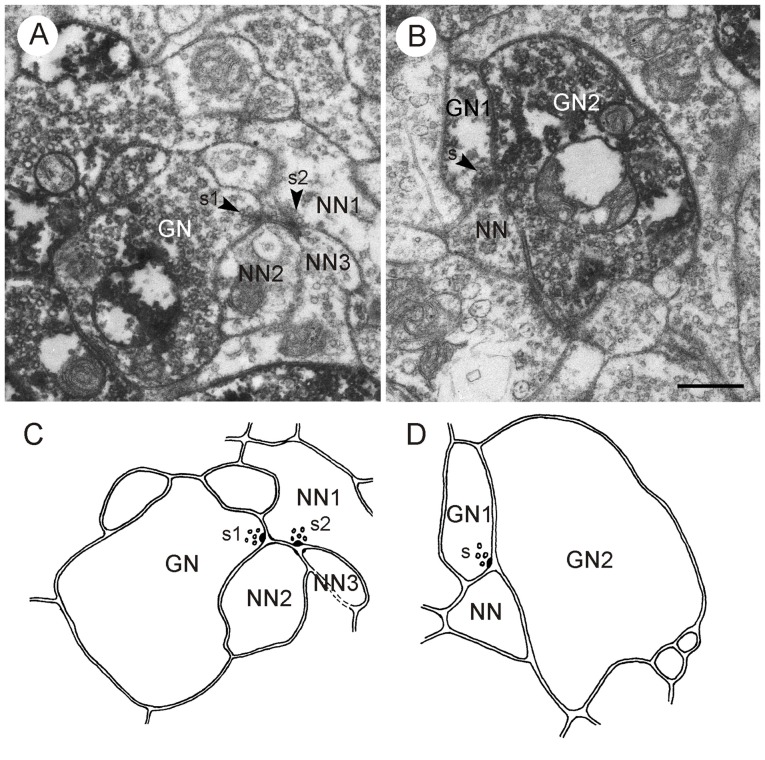
**GABA-immunoreactive neurons contributing to type I synapses; postembedding technique. (A,C)** Second out of two consecutive sections through the CBL **(A)** and corresponding schematic diagram of synaptic contacts **(C)**. A GABA-immunoreactive profile (GN) is presynaptic (s1, arrowhead) to two postsynaptic profiles (NN1,NN2). NN1 is, in addition, presynaptic (s2, arrowhead) to NN2 and a third unlabeled profile (NN3), illustrating serial connectivity. **(B,D)** Second out of two consecutive sections through the CBL **(B)** and corresponding schematic diagram of synaptic contacts **(D)**. A GABA-immunoreactive profile (GN1) is presynaptic (s, arrowhead) to a second GABA-labeled profile (GN2) and an unlabeled profile (NN). Scale bar in **(B)**: 0.5 μm (applies to **A–D**).

**Figure 9:**
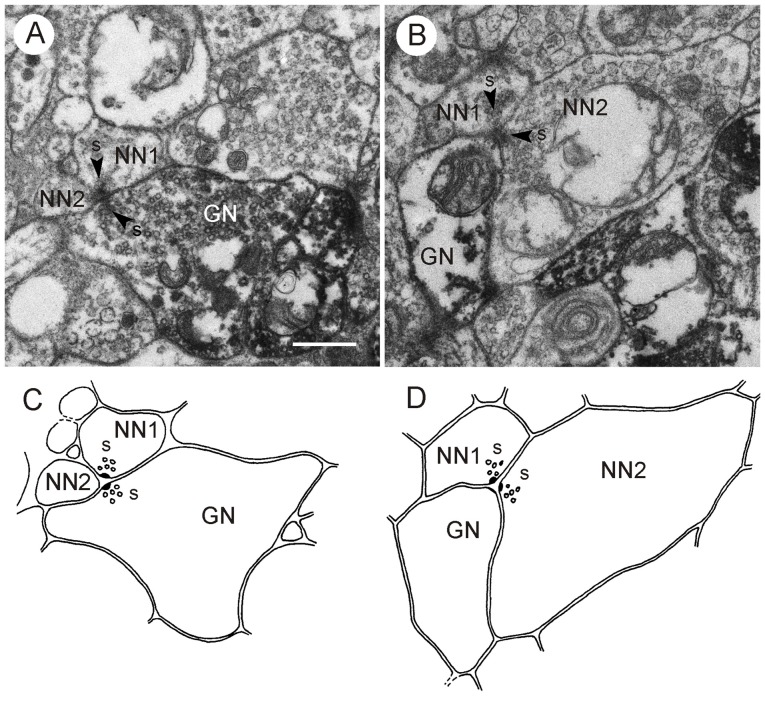
**GABA-immunoreactive neurons contributing to type II synapses; postembedding technique. (A,C)** Second out of two consecutive sections through the CBL **(A)** and corresponding schematic diagram of synaptic contacts **(C)**. A GABA-immunoreactive neuron (GN) and an unlabeled neuron (NN1) are presynaptic (s, arrowhead) to a second unlabeled profile (NN2). **(B,D)** Second out of two consecutive sections through the CBL **(B)** and corresponding schematic diagram of synaptic contacts **(D)**. A GABA-immunoreactive profile (GN) is postsynaptic to two unlabeled profiles (NN1, NN2) with presynaptic specializations (s, arrowheads). Scale bar in **(A)**: 0.5 μm (applies to **A–D**).

In contrast to the immunogold technique, GABA antiserum I was superior in the preembedding technique and was used for detailed evaluation of immunolabeling (Figures 8, 9). In preembedding GABA immunostaining, only low concentrations of Triton X-100 were used to achieve satisfactory preservation of ultrastructure. As a consequence, antisera penetration was impaired and therefore, only a small region below the surface of the Vibratome section was immunostained. Here, GABA-immunolabeled profiles could be easily distinguished from unlabeled neurons, based on the electron-dense diaminobenzidine precipitate.

Both staining techniques showed that GABA-immunolabeled profiles in the CBL were highly abundant. Although the numbers of labeled vs. unlabeled profiles were not evaluated quantitatively, we estimate that about 50% of all neuronal profiles in the CBL showed GABA immunostaining. In total, six profiles were identified as GABA-immunolabeled with the postembedding technique (Figures 5–7), and four using the preembedding technique (Figures 8, 9). These profiles had a size ranging from 0.3 μm^2^ (Figure 8B; GN1) to 5.6 μm^2^ (Figure 6). Prominent organelles were mitochondria and numerous vesicles. All GABA-immunolabeled profiles contained two different vesicle types, small clear vesicles and large dense core vesicles (Figures 5–7). The number of clear vesicles usually considerably exceeded that of the dense core vesicles (Figures 5–7). The clear vesicles were of pleomorphic appearance and were often indistinct, especially in comparison to small clear vesicles in profiles with numerous dense core vesicles. In addition, the content of the dense core vesicles in GABA-immunoreactive profiles was slightly less electron dense compared to dense core vesicles in profiles with numerous dense core vesicles. Profiles with large granular vesicles (Figures 5–7) and one profile with numerous dark dense core vesicles (not shown) were unlabeled.

### Synaptic Contacts of GABA-Immunolabeled Profiles in the CBL

GABA-immunolabeled profiles in the CBL had both input and output synapses with neighboring profiles. Nine of the 12 evaluated profiles had output synapses (Figures 5–9), four profiles received synaptic input (Figures 5, 7, 9). Two profiles had input and output synapses (Figures 5, 7). In total, 19 output synapses and six input synapses were found.

### Output Synapses

Three profiles contributed to all types of output synapses. In most cases the GABA-immunoreactive profile was presynaptic to two postsynaptic profiles (type I synapse; *n* = 13; Figures 5–8). In addition, configurations with one presynaptic GABA-immunostained profile, another unlabeled presynaptic profile, and a third unlabeled postsynaptic profile occurred (type II synapse; *n* = 6; Figures 5, 9). In both types of synapse, the postsynaptic profiles were usually much smaller than the presynaptic GABA-immunoreactive profiles (Figures 5, 6, 8). Their immunolabeling (GABA-positive or -negative) could, therefore, not be determined unequivocally in the immunogold preparations. Throughout the series of sections, however, most of these profiles had no or only one or two GPs, suggesting that they were not GABA-immunolabeled. Figure 8A shows a GABA-immunostained profile obtained by the preembedding technique which makes an output synapse with two clearly unlabeled profiles. Postsynaptic profiles often contained small clear vesicles, a few dark dense core vesicles or both (Figures 5–7). Very small profiles contained no vesicles at all (profile A in Figure 6). In two cases, postsynaptic profiles had large granular vesicles (Figure 6). In type II synapses, the second presynaptic profile was also GABA-immunoreactive in one case (Figure 5); in another case, the second presynaptic profile was not GABA-labeled (Figure 9A).

Three out of the eight identified GABA-immunoreactive profiles had conspicuously many output synapses (*n* = 4, resp. 5; Figures 5, 6). In all of these cases the neuron profile occupied a rather large area (up to 5.6 μm^2^), and these profiles had numerous vesicles and mitochondria. The postsynaptic profiles were largely small (<0.02 μm^2^) and arranged around the GABA-immunoreactive profile (Figures 5, 6). Some of these postsynaptic profiles received dual synaptic input from the same GABA-immunoreactive profile (profiles A and C in Figure 5; profiles B and C in Figure 6). One of the input synapses of profile A in Figure 5 is a type II synapse. In this case the second presynaptic profile is also GABA-immunoreactive. These configurations suggest that the small postsynaptic profiles in the CBL received massive GABAergic input.

Two instances showed a serial synaptic configuration in which a GABA-immunolabeled neuron contributed an output synapse (Figure 8A). The GABA-immmunolabeled profile in Figure 8 was presynaptic to two unlabeled profiles (NN1, NN2), and NN1 was itself presynaptic to NN2 and a third unlabeled profile NN3. A similar connectivity was found in another preparation (not shown).

### Input Synapses

Only five of the 12 evaluated GABA-immunolabeled profiles were postsynaptic to other profiles. Figures 7B,D shows an immunolabeled profile which receives triple synaptic input (s1–s3) from a non-classified profile with small clear vesicles and a few dense core vesicles. Two of these synaptic profiles (s1 and s3) had identical postsynaptic partners B and GN. The GABA-immunoreactive profile of Figure 7A,C received input via type I synapses from two different profiles. One of these profiles (NN, synapse s1) contained large granular vesicles. Although its cross sectional area was smaller than 0.5 μm^2^ it was most probably not GABA-immunoreactive, because it had no GP in any of the examined sections and many other profiles with similar vesicle content were shown to be immunonegative. The second presynaptic profile (A, synapse s2) contained dense core vesicles and small clear vesicles. Its immunolabeling could not be classified. The postsynaptic GABA-immunoreactive profile was itself presynaptic to two other non-classified profiles (C and D), illustrating serial synaptic contacts with the GABA-immunostained neuron being pre- and postsynaptic to surrounding profiles. In two type I synapses, GABA-immunoreactive profiles (GN2 in Figure 5 and GN2 in Figure 8B) received synaptic input from another GABA-immunostained profile (GN1 in Figures 5, 8B), which was also presynaptic to a third profile, which in Figure 8B was not GABA-immunolabeled. Figures 9B,D, finally shows a type II synapse with two non-immunolabeled neurons being presynaptic to a GABA-immunolabeled profile.

### LomTK Immunostaining

**Figure 10:**
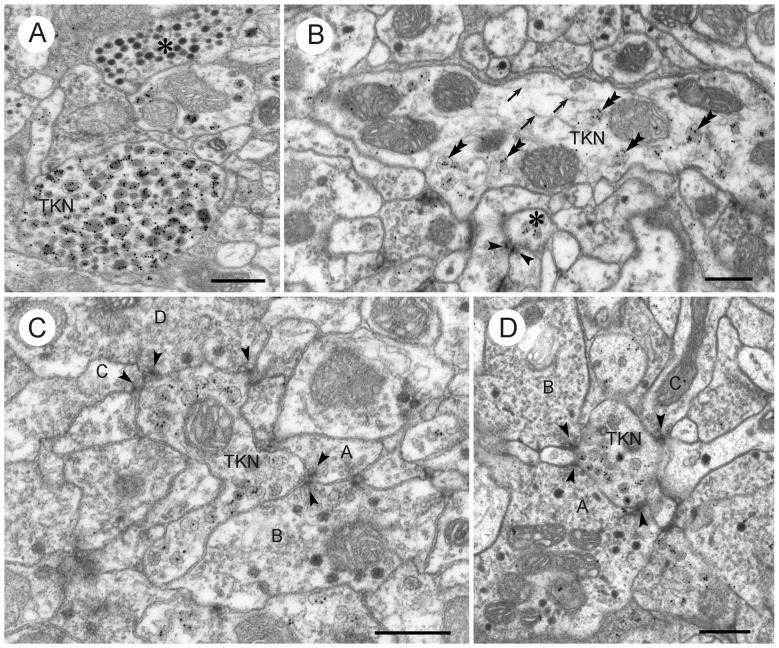
**LomTK II-immunoreactive neurons in the CBL. (A)** GPs are associated with granular dense core vesicles within a profile (TKN), but not with dark dense core vesicles in another profile (asterisk). **(B)** A larger fiber contains numerous microtubules (arrows), mitochondria (M) and a few granular vesicles. GPs are found almost exclusively on the granular vesicles (double arrowheads). A second small LomTK-labeled profile receives input from two neurons via a type II dyad. Arrowheads point to presynaptic densities. **(C,D)** Small LomTK-labeled profiles (TKN) receive numerous input synapses (type I and type II dyads) from unlabeled profiles (A–D in **C**, A–C in **D**). Arrowheads point to presynaptic densities. All of the presynaptic profiles contain small pleomorphic clear vesicles and in some of these (B,D in **C**; A,B in **D**) dark dense core vesicles are also present. Scale bars: 0.5 μm.

**Figure 11:**
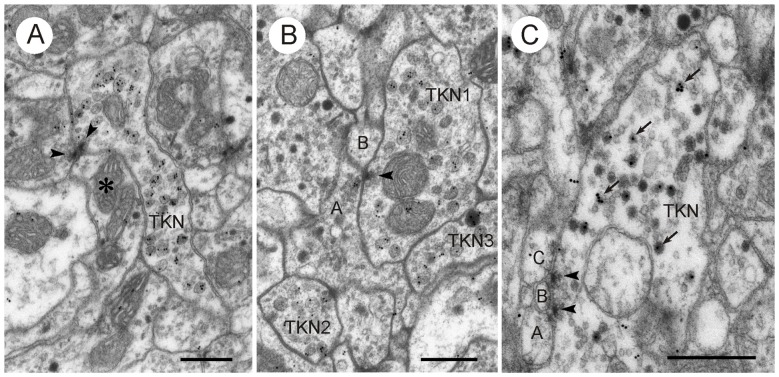
**LomTK II-immunoreactive neurons in the CBL. (A,B)** Profiles with LomTK-immunolabeled granular dense-core vesicles (TKN) making output synapses. **(A)** The immunolabeled profile is one of two presynaptic elements in a type II dyadic connection with a profile containing pleomorphic clear and a few dense core vesicles (asterisk). Arrowheads point to presynaptic densities. **(B)** Synaptic connection of an immunolabeled profile (TKN1) with one or two postsynaptic elements (A,B). Profile A contains pleomorphic clear and dark dense core vesicles. Arrowhead points to synaptic density. Two other profiles are LomTK-immunolabeled (TKN2, TKN3). **(C)** Profile (TKN) with small clear and large dark dense core vesicles. GPs are associated with the dense core vesicles (arrows). The profile makes two type I output synapses with three unclassified small profiles (A–C). Arrowheads point to presynaptic densities. Scale bars: 0.5 μm.

In contrast to GABA immunolabeling, immunogold staining for LomTK II revealed a strong association of GPs with a particular type of vesicle, large granular dense core vesicles (Figures 10, 11). This is particularly obvious in larger fibers, where granular dense core vesicles occurred more rarely (Figure 10B). In fact, granular vesicle containing profiles that were not immunostained were observed only very rarely. Based on GP density, immunolabeled profiles could be easily distinguished from unlabeled profiles, which rendered statistical analysis unnecessary (Figures 10, 11). Profiles with numerous dark dense core vesicles were unlabeled (Figure 10A). Likewise, profiles with dense core vesicles and pleomorphic clear vesicles were unlabeled except for two very rare cases, in which the GPs were found on dense core vesicles, but not on the clear vesicles (Figure 11C). Omission of the primary antiserum abolished all immunogold labeling on the sections.

LomTK-immunolabeled profiles had pre- and postsynaptic contacts with adjacent profiles. Small immunolabeled profiles were often surrounded by presynaptic profiles with pleomorphic clear and dense core vesicles which made both type I and type II synapses onto the LomTK profiles (Figure 10C,D). Output synapses more often occurred in larger profiles, again with postsynaptic partners that could often be identified as profiles with pleomorphic small clear vesicles and some large dense core vesicles. In several cases, output synapses had a monadic appearance, but adjacent sections for closer inspection were not available in those cases. In contrast to GABA-immunostained profiles, input and output synapses were never found together in the same profile. Likewise, synaptic contacts between two LomTK-immunolabeled profiles were not found.

## Discussion

The CBL in the insect brain is a major site of bilateral convergence of visual pathways from the two compound eyes (Homberg et al., 2011; Pfeiffer and Homberg, 2014). In the monarch butterfly, the field cricket, two species of dung beetle and the desert locust, neurons connecting the bulbs to the CBL, termed TL neurons, are sensitive to the plane of zenithal polarized light and constitute the principal input for sky compass signaling in the central complex (Vitzthum et al., 2002; Sakura et al., 2008; Heinze and Reppert, 2011; el Jundi et al., 2015). In the fly *Drosophila*, homologous neurons to the ellipsoid body are sensitive to the azimuth of a vertical light bar, and likely provide spatial landmark information for head direction coding in the central complex (Seelig and Jayaraman, 2013, 2015). In locusts and probably other species as well, two subtypes of TL neurons (TL2, TL3, Figure 1C) are GABA-immunoreactive suggesting that they signal through synaptic inhibition (Homberg et al., 1999). Candidate postsynaptic partners of TL neurons are columnar neurons (CL neurons) that occur in sets of 16 individuals and connect the CBL to the slices of the PB (Figure 1D). Two sets of CL1 neurons are LomTK-immunoreactive (Vitzthum and Homberg, 1998). How these different cell types are interconnected is unknown. The present study shows that the CBL in the desert locust is a neuropil of rich synaptic connectivities. Neuronal profiles differing distinctly in vesicle composition could be distinguished. Two types of synaptic contacts occur, convergent (type I) and divergent (type II) dyadic contacts. Both GABA- and LomTK-immunolabeled profiles show characteristic ultrastructures. GABA-immunostained neurons contain small clear and large dense core vesicles. They make predominantly output synapses to unlabeled profiles but, in addition, also receive synaptic input. LomTK immunostaining, in contrast, is largely confined to profiles with granular dense-core vesicles. LomTK-immunolabeled neurons make input and output synapses with unlabeled profiles, often those presumed to be GABAergic. The data support synaptic transmission from TL2/3 neurons to columnar CL1 neurons but suggest that CL1 neurons may also signal from the PB to the CBL. Both TL and CL neurons may, furthermore, be involved in local interactions in the CBL.

### Neuronal Organization and Ultrastructure of the CBL

The lower division of the locust central body is composed of the processes of at least seven types of tangential neuron and two types of columnar neuron (Müller et al., 1997; Heinze and Homberg, 2008; Bockhorst and Homberg, 2015). Tangential neurons, termed TL1–6 and TLU1 ramify in distinct areas of the lateral complex and target certain (TL2, TL3, TL4) or all layers (TL1, TL5, TL6, TLU1) of the CBL. Columnar neurons (CL1a-d, CL2) occur as systems of 16 neurons with ramifications in columnar domains of the lower division and projections to the PB, noduli (CL2) or LAL (CL1a, b, d). Similar cell types have been found in other insect species, notably, the monarch butterfly (Heinze et al., 2013) and the fruit fly *Drosophila* (Hanesch et al., 1989; Martín-Peña et al., 2014; Wolff et al., 2015). In sections largely from the center of the CBL, three types of vesicle could be distinguished in neuronal profiles, small clear vesicles, large dark dense core vesicles and large granular dense core vesicles. While clear vesicles have been associated with classical transmitters such as glutamate, GABA or acetylcholine, dense core vesicles have been shown to contain neuropeptides or biogenic amines (reviewed by Watson and Schürmann, 2002). Dyadic synaptic contacts as found here are the most common synaptic configurations in the nervous system of locusts (Schürmann and Wechsler, 1970; Leitch and Laurent, 1996; Watson and Schürmann, 2002; Träger et al., 2007) and other insect species (e.g., Tolbert and Hildebrand, 1981; Boeckh and Tolbert, 1993; Reischig and Stengl, 2003). In the CBL of *Drosophila* and the honeybee, in contrast, single (monadic) synapses were reported most frequently (Martín-Peña et al., 2014). These authors also found multiple synapses with two active zones in close proximity and coincident synapses with two presynaptic profiles facing a single postsynaptic profile. The latter type synapse probably corresponds to the type II (convergent) dyads found here. Convergent dyads are less common than type I (divergent) dyads, but have also been reported in other areas of the locust brain (Schürmann and Wechsler, 1970; Goodman et al., 1979; Leitch and Laurent, 1996). Martín-Peña et al. (2014) interpret these as coincidence detectors, possibly involved in learning and memory or decision making, but further studies are clearly required to substantiate these claims.

### GABA-Immunoreactive Neurons

The ultrastructure of GABA-immunoreactive neurons has been investigated in the brain and ventral nerve cord of various insect species (locust: Watson, 1988, 1990; Watson and Laurent, 1990; Leitch and Laurent, 1996; honeybee: Ganeshina and Menzel, 2001; cockroach: Distler, 1990; Malun, 1991). In all of these studies, GABA-immunolabeled profiles were characterized by the presence of numerous small pleomorphic clear vesicles and, often, smaller numbers of large dense core vesicles. The abundance of GABA-immunolabeled profiles in the locust CBL corresponds well with the dense innervation by an estimated number of about 100 bilateral pairs of TL neurons, which were identified as TL2, TL3 and TL4 (Homberg et al., 1999). The presence of large dense core vesicles in GABA-immunostained profiles suggests that these neurons contain peptide- or amine-cotransmitters. Candidate neuropeptides are peptides related to Dip-allatostatin, FMRFamide and orcokinin in TL4 neurons and LomTK II and orcokinin in TL2 neurons (Homberg et al., 1999; Hofer et al., 2005). A small subset of TL2 neurons, moreover, shows NADPH-diaphorase activity and may therefore release the gaseous transmitter nitric oxide (Kurylas et al., 2005). The high incidence of profiles with small clear vesicles and large dense core vesicles near the dorsal rim of the CBL, corresponding to the dorsalmost layer 1 (Figure 2C) likely relates to prominent immunostaining of TL4 neurons that exclusively innervate this layer for GABA, Dip-allatostatin, FMRFamide and orcokinin (Homberg et al., 1999; Hofer et al., 2005).

### LomTK Immunolabeling

At the light microscopic level, LomTK immunostaining in the CBL could be assigned to two sets of 16 CL1 neurons, corresponding to two neurons per slice of the CBL, and to two bilateral pairs of TL2 neurons (Vitzthum and Homberg, 1998). Double immunolabeling showed that the two TL2 neurons contain colocalized LomTK- and GABA-immunoreactivities (Vitzthum and Homberg, 1998). The ultrastructural data correspond well to the light-microscopic identification of immunolabeled neurons. LomTK immunogold labeling was highly concentrated on granular dense core vesicles, which suggests that profiles containing granular vesicles are those of the immunostained CL1 neurons. The much rarer finding of GP association with dark dense core vesicles in profiles with dense core and small clear vesicles (Figure 11C) is consistent with those profiles originating from the two LomTK/GABA-immunolabeled TL2 neurons. The regular presence of classical dyadic synapses in LomTK-labeled profiles suggests that, in addition to LomTK, these neurons also contain a classical neurotransmitter. So far, the ultrastructure of tachykinin-containing neurons has only rarely been investigated in the nervous system. In the vertebrate dorsal horn, tachykinin immunoreactivity is associated with large dense core vesicles (Merighi et al., 1991; Salio et al., 2001), and except for an early study on the locust brain (Benedeczky et al., 1982), no ultrastructural studies have been performed in invertebrates.

### Synaptic Connectivities and Functional Implications

Ultrathin sections of the CBL showed frequent synaptic contacts between different profiles usually in the form of divergent and, less frequently, convergent dyads. GABA-immunolabeled profiles had a higher number of output synapses compared to input synapses, suggesting that TL2, TL3 and TL4 neurons largely provide synaptic input to the CBL. This is consistent with the study of Träger et al. (2007), showing that TL2 and TL3 neurons have exclusively input synapses in the medial and lateral bulbs (LBUs; see Figure 1E). Output synapses in the CBL are made with a variety of profiles, including those containing large dense granular vesicles, presumed to originate from LomTK-labeled CL1 neurons. Other profiles may be those of CL2 neurons or LomTK-negative CL1 neurons. Synaptic contacts are also made between GABA-immunolabeled profiles suggesting lateral mutual inhibitions perhaps serving a role in shaping their tuning to polarized light. In addition, GABA-immunolabeled profiles also receive synaptic input in the CBL and often, output and input synapses were observed on the same profile. This suggests local interactions with unknown synaptic partners, possibly columnar neurons or TL1, TL5 or TL6 tangential neurons.

In LomTK-immunoreactive profiles, both input and output synapses were observed. Often, small LomTK-labeled profiles received numerous input synapses from surrounding profiles that often had the appearance of GABA-labeled ones, characterized by pleomorphic clear vesicles and small numbers of large dense core vesicles (Figures 10C,D). Here, the LomTK-immunolabeled CL1 neurons possibly receive synaptic input from polarized-light sensitive TL neurons. Output synapses in LomTK profiles occured usually individually and never together with input synapses on the same profile. This could mean that two different sets of LomTK-labeled profiles exist, one with output synapses and one with input synapses in the CBL, or that in the same neuron, input and output synapses as spatially segregated. Light microscopic observations, indeed, suggested that the tree of ramifications of CL1a neurons in the CBL is spatially organized into a core of stout varicose processes covering a single slice surrounded by a peripheral area of finer processes in adjacent slices (Heinze and Homberg, 2008). An ultrastructural analysis of single dye filled CL1 neurons might help to solve this question and bring more light into the synaptic connectivities of CL1 neurons in the CBL.

The present study supports electrophysiological data showing that CL1 neurons mainly receive inhibitory synaptic input from presynaptic neurons (Bockhorst and Homberg, 2015), likely to be the GABAergic TL2 and TL3 inputs to the CBL. The interactions between tangential and columnar neurons in the CBL is clearly an important step in shaping the topographic representation of preferred *E*-vector orientations in the slices of the PB, which occurs through neural computations and is not present at the level of TL neurons. An analysis of synaptic partners of individually labeled elements of the CBL is likely to shed further light on the synaptic computations in the CBL that contribute to the unique sensory representation in the PB.

## Ethics Statement

All animal procedures were in compliance with the guidelines of the European Union (Directive 86/609/EEc) and the German Animal Welfare Act. Approval of the study by an ethics committee was dispensable, because all experiments were on insects.

## Author Contributions

UH and MM conceived the research topic, MM performed most of the experiments, MM and UH evaluated the data, UH wrote the article.

## Funding

Supported by Deutsche Forschungsgemeinschaft (DFG) grant HO 950/24-1.

## Conflict of Interest Statement

The handling Editor declared a past co-authorship based on data generated 7 years ago, though no other collaboration, with one of the authors UH and states that the process nevertheless met the standards of a fair and objective review. The other author declares that the research was conducted in the absence of any commercial or financial relationships that could be construed as a potential conflict of interest.
